# Assessment of Obesity as Risk Factor of Lumbar Disc Surgery: Retrospective Analysis of 598 Cases and Simulated Surgery on 3D-Printed Models

**DOI:** 10.3390/jcm13144193

**Published:** 2024-07-18

**Authors:** Ralf Stroop, Fernando Carballar, Samer Zawy Alsofy, Hraq Sarkis, Makoto Nakamura, Christoph Greiner, Bernhard Dorweiler, Moritz Wegner

**Affiliations:** 1 Faculty of Health, Department of Medicine, Witten-Herdecke University, 58455 Witten, Germany; ralf.stroop@uni-wh.de (R.S.); fercarballar90@gmail.com (F.C.); szawyalsofy@barbaraklinik.de (S.Z.A.); sarkis.hraq@klinikum-luenen.de (H.S.); 2 Medical School Hamburg, 20457 Hamburg, Germany; 3 Niels Stensen Neuro Center, Department of Neuro- and Spine Surgery, 49076 Osnabrück, Germany; christoph.greiner@niels-stensen-kliniken.de; 4 St. Marien Hospital Lünen, Academic Teaching Hospital of University of Münster, 44534 Lünen, Germany; 5 Department of Neurosurgery, Academic Hospital Cologne-Merheim, Witten-Herdecke University, 51109 Cologne, Germany; nakamuram@kliniken-koeln.de; 6 Department of Vascular and Endovascular Surgery, Faculty of Medicine and University Hospital Cologne, University of Cologne, 50937 Cologne, Germany; bernhard.dorweiler@uk-koeln.de

**Keywords:** obesity, spinal surgery, lumbar disc herniation, 3D printing, simulation, simulated surgery

## Abstract

**(1) Background**: Obesity poses known risks in surgery, including a prolonged operation time and postoperative complications. Given the rising obesity rates and frequent lumbar disc surgeries, understanding these risks is crucial. This study aims to assess the impact of obesity on operation duration and postoperative complications in lumbar disc prolapse surgery. **(2) Methods**: We retrospectively analyzed 598 patients with monosegmental disc herniation, correlating their body mass index (BMI) as a surrogate parameter for obesity with operation time. Excluding complex cases (multi-segmental herniations or recurrent herniations), complication rates and hospital stays were recorded. Simulated surgeries on 3D-printed models of varying obesity levels examined operation times and instrument suitability. **(3) Results**: Of these patients, 438 patients had a BMI of <30, and 160 patients had a BMI of ≥30. Complication rates showed no significant differences between groups. Linear regression analysis failed to establish a sole dependency of operation time on BMI, with R^2^ = 0.039 for the normal-weight group (BMI < 30) and R^2^ = 0.059 for the obese group (BMI ≥ 30). The simulation operations on the 3D-printed models of varying degrees of obesity showed a significant increase in the simulated operation time with higher levels of obesity. A geometrically inadequate set of surgical instruments was assumed to be a significant factor in the simulated increase in operating time. **(4) Conclusions**: While various factors influence operation time, obesity alone does not significantly increase it. However, simulated surgeries highlighted the impact of obesity, particularly on instrument limitations. Understanding these complexities is vital for optimizing surgical outcomes in obese patients.

## 1. Introduction

Surgical access to the lumbar spine ranks third in Germany in terms of the frequency of all operative procedures performed [1]. Concurrently, the World Health Organization (WHO) has reported a nearly threefold increase in the prevalence of obesity since 1975 [2]. Obesity, beyond its association with comorbidities such as diabetes mellitus, hypertension, or mental disorders like depression, is also implicated in a heightened incidence of disc degeneration, spinal arthritis, and lower back pain [3,4]. Collectively, these factors suggest a potential increase in spinal surgery for obese patients in the future. While obesity alone poses a significant risk for wound infection, increased surgical blood loss, and an extended operative time in general surgery [5], its impact on spinal surgery remains a subject of controversy [6]. Existing evidence indicates a prolonged surgery time in severely obese patients with disc herniation [7], and in obese patients, the clinical benefits and outcomes of both surgical and nonsurgical treatments for lumbar disc herniation may be diminished [8]. Conversely, some studies suggest that obesity does not necessarily predict poorer symptom control and quality of life after lumbar disc surgery [9].

When determining the indication for surgery in patients with lumbar disc herniation (LDH), it is important to note that, as demonstrated by Gugliotta et al. [10], surgical intervention can lead to a faster improvement in symptoms compared to conservative treatment. However, it is also crucial to recognize that similar treatment outcomes can be achieved in midterm and long-term follow-ups with conservative management, thereby avoiding the risks associated with surgery.

To substantiate the relevance of the “obesity” factor in the decision or indication for the conservative or surgical treatment of herniated discs, this study was conducted with the hypothesis that the body mass index (BMI), as a surrogate parameter of obesity, directly influences operation time. Secondarily, we aimed to explore the extent to which the BMI might impact the intra- and postoperative complication rates, as well as the length of hospital stay for patients undergoing monosegmental disc surgery in the lumbar spine.

Recent advancements in three-dimensional (3D) printing technology have significantly impacted various fields, including medical research and practice. The use of 3D-printed models has proven beneficial in pre-surgical planning, education, and the development of patient-specific implants and prosthetics [11,12,13]. According to recent studies, 3D printing allows for the creation of accurate and customizable anatomical models that can enhance the precision of surgical procedures and improve patient outcomes [14]. “Bioadditive manufacturing” and “bioprinting” have further expanded these capabilities, enabling the fabrication of complex biological structures and tissues, which hold promise for regenerative medicine and advanced surgical training [15]. These technologies provide a versatile and cost-effective solution for replicating complex structures and have been increasingly integrated into clinical workflows. The evolution of 3D printing from basic prototyping to the development of sophisticated, functional models underscores its potential in transforming surgical practices and advancing personalized medicine. This study aims to assess the application of 3D-printed models in lumbar disc surgery, exploring their utility in simulating surgical scenarios.

Consequently, we retrospectively evaluated the operation times for patients with monosegmental disc herniation based on their degree of obesity, considering various factors influencing surgical time. Additionally, we conducted surgical simulations on differently configured, MRI-based 3D-printed models to assess obesity as a potential risk factor for lumbar disc surgery.

## 2. Materials and Methods

### 2.1. Evaluation of Obesity-Dependent Complications and Surgery Duration

Following approval from the local ethical committee (IRB S-37/2024), we conducted a thorough screening of our institutional database to identify patients who underwent initial monosegmental disc surgery in the lumbar spine using microsurgical techniques between July 2016 and July 2020 at a single center for retrospective analysis. We hypothesized that an increased BMI, as a surrogate for obesity, may be associated with an increase in operating time. The STROBE statement for observational studies was followed.

The exclusion criteria encompassed cases of recurrent disc herniation, multi-segmental surgery, the necessity for spondylodesis, individuals below the age of 18, and those incapable of providing informed consent. These exclusion criteria were designed to pinpoint variables unrelated to the patient’s BMI that could potentially inflate or decrease operation time.

Data extraction was performed using the ORBIS electronic patient record system (Agfa HealthCare, Mortsel, Belgium). The gathered data encompassed patient demographics, including gender, age, and BMI. Additionally, pre- and post-operative clinical information such as paresis, cauda symptoms, post-operative hemorrhage, wound healing, and the length of hospital stay after surgery were recorded. In addition, important surgical criteria included in the analysis were the duration of the procedure in minutes, the surgical height, the orientation of the herniated disc, whether a nucleotomy was performed, the intraoperative occurrence of dura and nerve injuries, and the identity of the surgeon who performed the procedure.

### 2.2. Creation of 3D-Printed Models

We systematically screened our institutional database for MRI scans of the lumbar spines from patients who had undergone decompressive surgery for disc protrusion or prolapse. We specifically selected four representative scans from patients with varying BMIs and differing subcutaneous fatty tissue thicknesses (Table 1). The chosen DICOM files were then imported into a dedicated 3D post-processing and engineering software (3D Slicer image computing platform, Version 4.11, open-source software, provided by the Brigham and Women’s Hospital, Boston, MA, USA) [16]. Subsequently, a semi-automatic segmentation process was employed to delineate bones and soft tissue.

In essence, we segmented the soft tissue volume, encompassing the subcutaneous tissue, intervertebral discs, and dural sac, along with the major bones of the lumbar spine, including vertebral bodies, spinous processes, and joints. We then formed a hollow channel, serving as a preformed access point to the spinal canal of the 3D model to enable the placement of retractors and instruments.

Subsequently, a 3D file (.stl) was generated and printed using a high-resolution PolyJet-technology 3D printer (Objet 350 Connex3, Stratasys, Rehovot, Israel). The 3D model included both flexible components (representing soft tissue) and solid elements (representing bones). Agilus^®^30, a liquid photopolymer with a proprietary composition, polymerized by UV light-curing, was utilized for the soft tissue (density (polymerized) 1.15 g/cm^3^, tensile strength 2.4–3.1 MPa, hardness (ShoreA) 30–35; Stratasys, Rehovot, Israel) and VeroWhite^®^ was used for the bones (density (polymerized) 1.18 g/cm^3^, tensile strength 50–65 MPa, hardness (ShoreD) 83–86; Stratasys, Rehovot, Israel).

**Figure 1:**
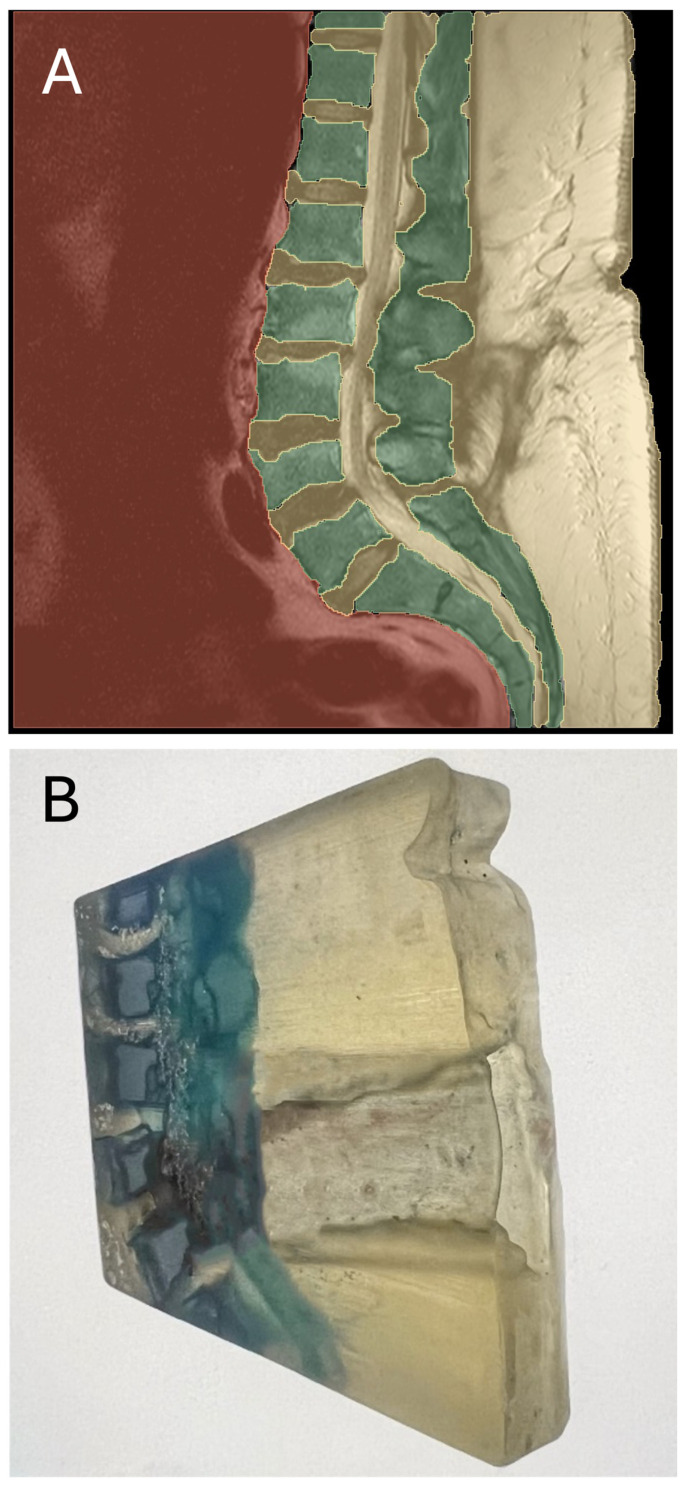

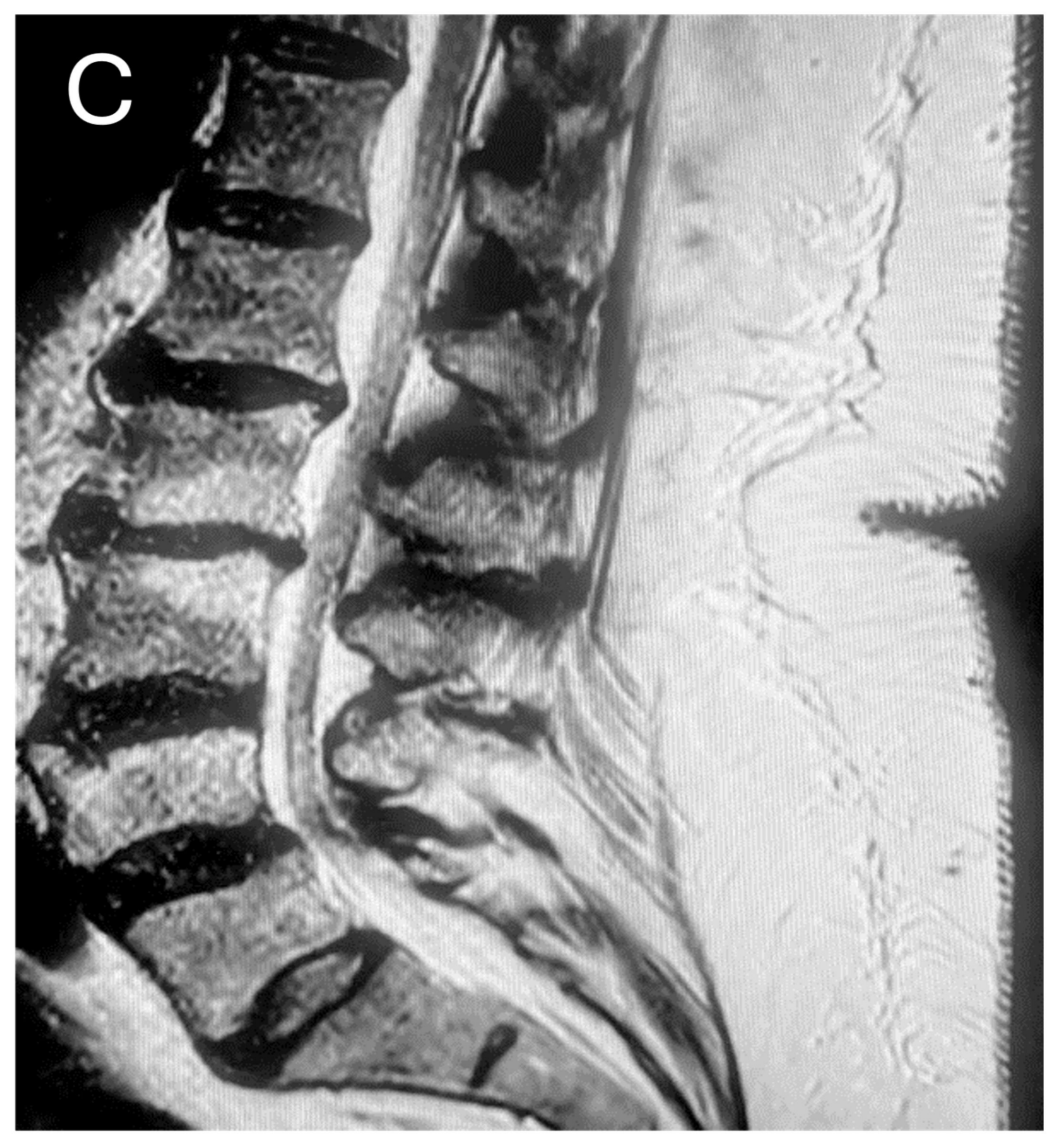
Three-dimensional slicer-based segmentation (**A**), 3D-printed model (**B**), and corresponding sagittal lumbar T2-weighted MRI scan (**C**). The patient suffered from an intraforaminal lumbar disc prolapse that was evident on more lateral slices, which are not presented here.

The printing resolution was set to maximum, with a layer resolution of 16 μm. Post-printing, a meticulous cleaning process was undertaken, including the removal of the support material. This process consistently produces 3D-printed models with a high anatomical fidelity, exhibiting minimal spatial deviation (±120 µm) [17]. An example of this process is depicted in Figure 1A–C.

### 2.3. Simulation of a Herniated Disc Operation

To assess the potential impact of obesity or surgeon-specific factors on operation times, simulations of herniated disc surgeries were conducted using the 3D-printed models. A total of five different models were generated, representing four distinct patients and accounting for variations in the depth of the situs. The key parameters are detailed in Table 1. Notably, models 4 and 5 were constructed based on magnetic resonance images from the same patient.

In a simulation closely mimicking real-world conditions, the models were positioned on the operation table of the operating theatre. Alongside a KINEVO 900 surgical microscope (Karl Zeiss, Jena, Germany), the setup included both a standard microsurgical tray designed for LDH operations and a bariatric operating tray (Aesculap, Tuttlingen, Germany). The microsurgical tray comprised various instruments such as nerve hooks (200 mm), Kerrison bone punches (180 mm), and grasping forceps (curved, length 150 mm; straight, 150–200 mm). Muscle retractors (CASPAR, Aesculap, Tuttlingen, Germany; depth 40–75 mm) were also part of this tray. The bariatric operating tray featured similar tools, including different nerve hooks (200 mm), Kerrison bone punches (200–250 mm length), grasping forceps (curved, length 150–230 mm; straight, 150–280 mm), and muscle retractors (CASPAR, Aesculap, Tuttlingen, Germany; depth 40–100 mm).

Ten neurosurgeons, each with substantial experience in lumbar disc prolapse surgeries, were tasked with a series of steps during the simulation. The objective was to (1) appropriately insert a suitable retractor into the models, arranged in ascending order according to their degree of obesity, (2) aspirate a colored saline fluid with the aspirator, (3) manipulate a small tissue fragment using the nerve hook, (4) excise a portion of it with the punch, and (5) retrieve the excised tissue with the grasping forceps.

### 2.4. Questionnaire-Based Assessment of the Simulation Process

To gauge the realism of the simulation and assess the effectiveness of the available surgical instruments, a comprehensive questionnaire was employed.

### 2.5. Statistics

Data were statistically analyzed using the SPSS software package (version 28.0, IBM Corp., Armonk, NY, USA). The normality of the data was assessed using the Shapiro–Wilk test. Data are expressed as median with interquartile range (IQR) for nonparametric data and as mean with standard deviation for parametric data. The Mann–Whitney test for independent and Wilcoxon signed-rank test for dependent samples were used to compare continuous variables, and the chi-square test was used to compare categorical variables. One-way analysis of variance (ANOVA) was used to determine whether a relationship existed between more than two variables. Linear regression and multivariate correlation analyses were performed to evaluate the factors potentially influencing the operation time in addition to BMI, and the intraclass correlation coefficient was used to determine inter-rater reliability. A *p* value of <0.05 was regarded as significant.

## 3. Results

### 3.1. Evaluation of Obesity-Dependent Complications and Surgery Duration

Out of the 598 patients meeting the specified inclusion criteria, n = 438 had a BMI less than 30, while n = 160 had a BMI of 30 or more. Among these patients, a balanced gender distribution was observed, with n = 87 males (54%) and n = 73 females (46%). Their demographics are shown in Table 2.

The incidence of intraoperative complications, including dura leakage (3.9% vs. 2.5%, *p* = 0.223), post-operative hemorrhage (0.7% vs. 1.9%, *p* = 0.092), paresis (0.5% vs. 0.6%, *p* = 0.969), and cauda equina syndrome (0.5% vs. 1.3%, *p* = 0.770), in relation to the degree of obesity measured by BMI, revealed no statistically significant distinction between the two BMI groups (<30 and ≥30). Additionally, the groups did not differ regarding the length of hospital stay, with a median (IQR) stay of 3 (2–3) days in both groups (*p* = 0.326). However, univariate analysis showed a prolonged surgery duration in patients with a BMI of 30 or more, with median (IQR) durations of 51 (34–70) min in patients with a BMI under 30 and 60 (44–82) min in patients with a BMI of 30 or more (*p* < 0.001).

The consideration of additional factors that might potentially impact operation time, including the patient’s gender, segment height, herniated disc orientation (medial, mediolateral, intraforaminal, extraforaminal), whether a nucleotomy was performed, duroplasty application in cases of dura leakage, and the individual dexterity of the surgeon, revealed a co-explanatory effect of 44% for fluctuations in operation time, alongside obesity. This was determined by the calculation of the determination coefficient (R^2^). Linear regression analysis, however, failed to establish a sole dependency of operation time on BMI in either of the two groups, with R^2^ = 0.039 for the normal-weight group (BMI < 30) and R^2^ = 0.059 for the obese group (BMI ≥ 30).

Surgeries were performed by 13 surgeons in total and the mean individual operating times ranged from 26.9 (±10.2) to 92.9 (±27.11) minutes. A one-way ANOVA revealed statistically significant differences in the mean operating times between surgeons (F(12, 585) = [41.372], *p* < 0.001).

In subsequent subgroup analyses, the impacts of these individual factors were explored in conjunction with the BMI grouping. Apart from a moderate correlation (R^2^ = 0.338) between BMI and operating time in surgeries for intraforaminal disc herniation among female patients and a weak correlation between BMI and operating time in 5 out of 13 surgeons (R^2^ between 0.11 and 0.274), the linear regression analysis indicated that none of the other analyzed factors significantly influenced the operating time (Table 3). The dependency of the operation time on the surgeons did not appear to be contingent on their individual levels of experience.

### 3.2. Simulation of Surgeries using 3D-Printed Models

The 10 surgeons simulated the previously defined surgical steps using the provided surgical instruments, and the individual steps and total operating times were subjected to analysis.

The assessment of the total time required for the surgical simulation demonstrated a high level of interrater reliability, as indicated by the intraclass correlation coefficient (ICC) in the comparison of the 10 surgeons. The ICC values revealed a high or very high agreement of the required simulation time for all five models representing increasing obesity (Table 1).

**Figure 2:**
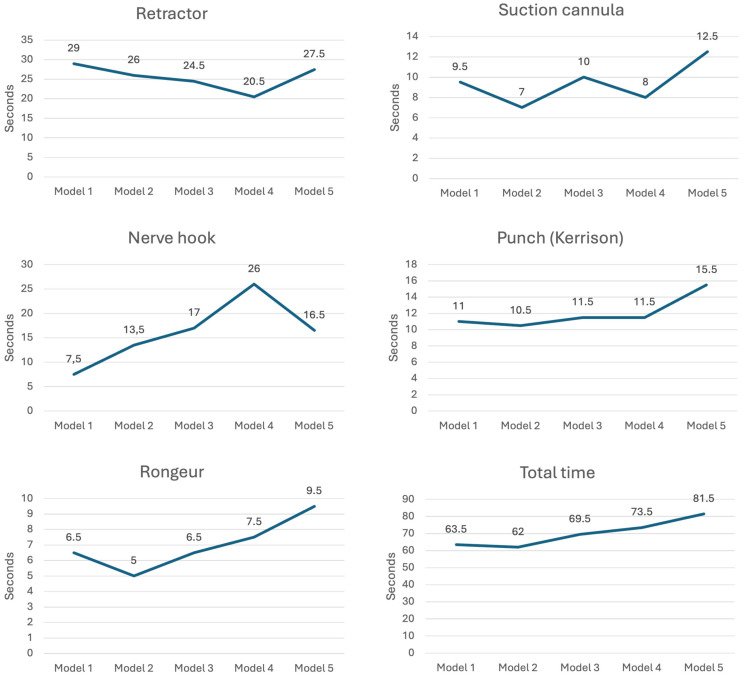
Mean surgical instrument utilization times for performing the individual tasks and total operating times depending on 3D-printed models representing increasing obesity.

The utilization times of surgical instruments for each individual task and the total operating times for the surgical simulation, depending on the five 3D-printed models representing increasing obesity, are illustrated in Figure 2. While there was a certain trend toward longer median utilization times of the instruments when performing individual simulated surgical steps with increasing adiposity models, a learning curve was apparent when using the retractor for models with higher adiposity levels. The total time demonstrated a significant increase with higher levels of obesity.

### 3.3. Questionnaire-Based Assessment of the Simulation Process

**Figure 3:**
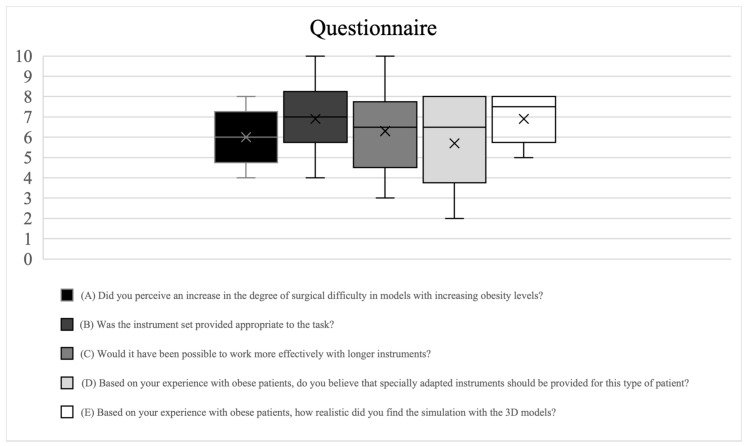
The responses to the questionnaire on the performance of the surgical simulations, showing the median of the responses of all 10 surgeons surveyed, the minimum and maximum values and the quartile intervals, in boxplot form with x = mean, horizontal line = median, 1st and 3rd quartile intervals.

The interpretations of the median values from the responses given by the 10 surveyed surgeons to the questionnaire are outlined below (Figure 3):

(A) A certain, but not too pronounced, increase in difficulty was reported toward the models representing a higher obesity level.
(B) The instruments were rated by the operators as ‘rather appropriate’, and, in some cases, ‘appropriate’ for carrying out the simulation tasks, even on the models representing a higher obesity level.
(C) Although all operators stated an increased effectiveness of longer instruments in carrying out the simulation tasks, the expected degree of increased effectiveness varied considerably.
(D) Although the need for longer instruments for surgery on obese patients was recognized, it was rated as rather moderate.
(E) The model was recognized as having a certain degree of realism, and the operators were relatively consistent in this assessment.
(F) All surgeons agreed that the nerve hook was the least suitable option, and three out of seven surgeons also mentioned the punch.

## 4. Discussion

To establish the relevance of obesity as a factor influencing the decision between the conservative or surgical treatment of herniated discs, we conducted a retrospective data collection correlating BMI with surgery time. Subsequently, using MRI-based 3D-printed situs models, we aimed to evaluate the geometric and steric suitability of surgical instruments for obese patients.

### 4.1. Evaluation of Obesity-Dependent Complications and Surgery Duration

Contrary to expectations, our data did not reveal a clear correlation between the surgery time and the degree of obesity. Additionally, there was no observed correlation between the frequency of surgery-related complications and the extent of obesity. This indicated that obese patients did not experience significantly more complications than non-obese patients during or after lumbar disc surgery. Notably, the influence of various factors on operation time could not be entirely eliminated. These included the complexity of the case, the specific technique used, and the condition of the patient. These factors may have contributed to the variability in the surgery times observed. Interestingly, when analyzing the R^2^-values on the association between body mass index and the operative time taken by different surgeons, there was remarkable variability in the strength of this association. Although the overall impact of BMI on operative time was limited and some experienced surgeons did not face significant challenges with obese patients, others experienced a notable increase in operating time. This variability suggests that individual surgeon experience and skill may play critical roles in the impact of obesity on surgery duration. Experienced surgeons might be better equipped to handle the challenges posed by obesity, leading to less variation in their surgery times. This underscores the importance of personalized approaches and continual skill development in surgical practice. Future research should continue to explore these dynamics to improve the surgical outcomes for different patient populations.

### 4.2. Simulation using 3D-Printed Models

To further explore the isolated influence of obesity on surgery time, we utilized 3D-printed models in surgical simulations. The tasks to be performed by the surgeons in simulating a prolapsed disc operation demonstrated a significantly longer operating time for models representing a higher level of obesity. Additionally, the simulations highlighted the limitations of surgical instruments, particularly in terms of geometric and steric considerations, with shorter tools proving suboptimal.

### 4.3. Significance of 3D-Printed Models

The increasing importance of 3D-printed situs models in surgical simulations cannot be overstated. Beyond our study, these models find utility in preoperative planning, providing surgeons with a detailed understanding of patient-specific anatomy derived from CT or MRI scans [18,19,20,21]. Furthermore, they contribute to medical education, offering a realistic platform for training and continuing medical education [11]. In patient education and consent processes, 3D models enhance understanding and compliance. In personalized medicine, 3D-printed implants hold promise for optimized fitting and effectiveness tailored to individual anatomy [22,23,24].

### 4.4. Addressing the Dilemma of Surgery for Herniated Discs

Surgery for herniated discs remains a complex decision, often presenting a dilemma, especially when high-grade paresis is absent. Conservative approaches entail prolonged pain symptoms, while surgery introduces inherent surgical risks. The existing literature offers contradictory statements, reporting prolonged surgery times for severely obese patients [7], lower clinical benefits regardless of surgical or nonsurgical treatment [8], and conflicting findings on the impact of obesity on symptom control and quality of life after lumbar disc surgery [9].

### 4.5. Limitations

Our study has inherent limitations. The retrospective data collection relied on BMI as a surrogate for obesity, lacking consideration for individual body fat distribution. The operation times were extracted from documentation rather than explicitly recorded. The regression analysis failed to show any difference between groups and a propensity matched score analysis was not attempted, as it would affect the representativeness of the cohort. Larger cohort studies could probably provide more information and permit the identification of additional confounders. Simulation examinations, while valuable, involved preformed 3D-printed models, and additional time expenditure related to obesity was simulated only during retractor insertion.

### 4.6. Future Research Directions

Due to the yearly increasing incidence of LDH in obese adolescents, further studies are recommended to evaluate the optimal treatment outcome. Not only a comparison of conservative and surgical treatment, but also a comparison of the surgical procedures with each other.

While only open microsurgical discectomy was examined in the present study, the results of Qu et al. from the evaluation of endoscopic lumbar discectomy in overweight versus normal-weight patient cohorts showed a significantly longer operative time, increased X-ray exposure, and longer time to ambulation, without a significant difference in complications, with the latter in accordance with our results [25].

In addition, a more subtle differentiation of obesity grading into BMI (kg/m^2^) < 20 (underweight), 20–25 (normal weight), 26–30 (overweight), 31–35 (obesity grade 1), and >35 (obesity grade 2), as outlined in the German Spine Register [26], should be considered in future studies with an adequate sample size. In order to capture the effect of the individual or gender-specific fat distributions of patients and to predict the obesity-associated risk more accurately, a newly defined assessment measure that approximates the extent of the back fat layer thickness, such as the distance from the skin to the inner angle of the arcus vertebrae, would be a variable with a significant influence on the access-related surgical risk. Instead of a BMI-value-based evaluation, the back fat layer thickness determined in this way would be an interesting parameter for comparing endoscopic and microsurgical open surgical procedures in terms of the operating time in a 3D model-based simulation.

The production and evaluation of video recordings and the use of motion tracking software during both real surgeries and simulated operations on 3D-printed models could help to track the individual movements of different surgeons to understand the differences in operation times more precisely [27].

### 4.7. Tissue Modeling Challenges

Attempting to precisely map tissue parameters in 3D, including damping, elasticity, and viscoelastic properties, remains a significant challenge. While various tissue models aim to describe physical behavior realistically [28], achieving accurate 3D representation is complex.

## 5. Conclusions

Our study, while not conclusively resolving the debate on surgery for obese patients with herniated lumbar disc prolapses, offers evidence that experienced surgeons may not invariably encounter a substantial increase in operating time. For spinal centers routinely conducting LDH surgeries in obese individuals, the establishment of a dedicated bariatric tray emerges as a pragmatic step to optimize patient care. This conclusion underscores the transformative impact of 3D printing in shaping surgical approaches and patient outcomes.

## Figures and Tables

**Table 1:** Segment height, surgical situs depth in mm, measured from the skin surface to the center of the intervertebral disc space, body mass index (BMI), and intraclass correlation coefficient (ICC) derived from linear correlation analysis of the five 3D-printed surgical situs models.

Model	1	2	3	4	5
Segment	LVB 4/5	LVB 4/5	LVB 4/5	LVB 4/5	LVB 5/SVB 1
Surgical Situs Depth (mm)	106	127	155	169	172
BMI (kg/m^2^)	32.1	32.7	52.6	44.2	44.2
ICC	0.973	0.940	0.912	0.931	0.879

LVB = lumbar vertebral body, SVB = sacral vertebral body, and ICC = intraclass correlation coefficient.

**Table 2:** Demographics of included patients.

	All Patients (n = 598)	BMI < 30 kg/m^2^ (n = 438)	BMI > 30 kg/m^2^ (n = 160)	*p*-Value
Age in years	50 (40–61)	51 (40–62)	48 (39–60)	0.143
Female gender	263 (44.0)	190 (43.4)	73 (45.6)	0.624
BMI (kg/m^2^)	26.9 (23.9–30.2)	25.0 (23.0–27.4)	32.8 (31.1–35.6)	<0.001

Data are presented as median (IQR) or n (%); BMI = body mass index.

**Table 3:** Linear correlation evaluation of factors potentially influencing operation time in addition to the body mass index.

				BMI < 30	BMI ≥ 30
All patients	All	all		0.039 (*p* = 0.001; n = 438)	0.059 (*p* = 0.002; n = 160)
		Age	<60	0.046 (*p* = 0.001; n = 302)	0.104 (*p* = 0.001; n = 117)
			≥60	0.027 (*p* = 0.035; n = 136)	0.008 (*p* = 0.057; n = 43)
Gender	Male	Age	<60	0.054 (*p* = 0.001; n = 176)	0.156 (*p* = 0.001; n = 66)
			≥60	0.087 (*p* = 0.005; n = 72)	0.11 (*p* = 0.141; n = 21)
	Female	Age	<60	0.041 (*p =* 0.08; n =126)	0.055 (p = 0.102; n = 51)
			≥60	0.01 (*p =* 0.792; n = 64)	0.02 (p = 0.846; n = 22)
Orientation	Medial/mediolateral			0.042 (*p* = 0.001; n = 363)	0.05 (*p =* 0.01; n = 135)
	Intraforaminal			0.014 (*p* = 0.421; n = 38)	0.261 (*p =* 0.242; n = 7)
	Extraforaminal			0.058 (*p* = 0.307; n = 13)	0.12 (*p =* 0.447; n = 7)
Segment	LVB 2/3			0.01 (*p* = 0.962; n = 20)	0.57 (*p =* 0.19; n = 9)
	LVB 3/4			0.045 (*p* = 0.147; n = 51)	0.062 (*p* = 0.29; n = 21)
	LVB 4/5			0.012 (*p* = 0.151; n = 179)	0.082 (*p* = 0.027; n = 70)
	LVB 5/SVB 1			0.02 (*p* = 0.576; n = 178)	0.043 (*p* = 0.127; n = 60)

R^2^ values represent the determination coefficient indicating the proportion of variability in operation time explained by the respective factor (significance parameter p; occurrence n). LVB = lumbar vertebral body and SVB = sacral vertebral body.

## Data Availability

The data presented in this study are available from the corresponding author upon request. The data are not publicly available due to restrictions.

## References

[1] Statistisches Bundesamt Die 20 Häufigsten Operationen Insgesamt (OPS5). https://www.destatis.de/DE/Themen/Gesellschaft-Umwelt/Gesundheit/Krankenhaeuser/Tabellen/drg-operationen-insgesamt.html.

[2] WHO Obesity and Overweight. https://www.who.int/news-room/fact-sheets/detail/obesity-and-overweight.

[3] Liuke M., Solovieva S., Lamminen A., Luoma K., Leino-Arjas P., Luukkonen R., Riihimäki H. (2005). Disc Degeneration of the Lumbar Spine in Relation to Overweight. Int. J. Obes..

[4] Gandhi R., Woo K.M., Zywiel M.G., Rampersaud Y.R. (2014). Metabolic Syndrome Increases the Prevalence of Spine Osteoarthritis. Orthop. Surg..

[5] Tjeertes E.E., Hoeks S.S., Beks S.S., Valentijn T.T., Hoofwijk A.A., Stolker R.J.R. (2015). Obesity—A Risk Factor for Postoperative Complications in General Surgery?. BMC Anesthesiol..

[6] Goyal A., Elminawy M., Kerezoudis P., Lu V.M., Yolcu Y., Alvi M.A., Bydon M. (2019). Impact of Obesity on Outcomes Following Lumbar Spine Surgery: A Systematic Review and Meta-Analysis. Clin. Neurol. Neurosurg..

[7] McGuire K.J., Khaleel M.A., Rihn J.A., Lurie J.D., Zhao W., Weinstein J.N. (2014). The Effect of High Obesity on Outcomes of Treatment for Lumbar Spinal Conditions: Subgroup Analysis of the Spine Patient Outcomes Research Trial. Spine.

[8] Rihn J.A., Kurd M., Hilibrand A.S., Lurie J., Zhao W., Albert T., Weinstein J. (2013). The Influence of Obesity on the Outcome of Treatment of Lumbar Disc Herniation: Analysis of the Spine Patient Outcomes Research Trial (Sport). J. Bone Jt. Surg..

[9] Brennan P.M., Loan J.J., Watson N., Bhatt P.M., Bodkin P.A. (2017). Pre-Operative Obesity Does Not Predict Poorer Symptom Control and Quality of Life after Lumbar Disc Surgery. Br. J. Neurosurg..

[10] Gugliotta M., da Costa B.R., Dabis E., Theiler R., Jüni P., Reichenbach S., Landolt H., Hasler P. (2016). Surgical versus Conservative Treatment for Lumbar Disc Herniation: A Prospective Cohort Study. BMJ Open.

[11] Wegner M., Dusse F., Beeser F., Leister N., Lefarth M., Finke S.R., Böttiger B.W., Dorweiler B., Stoll S.E. (2024). Comparing Simulation Training of Bronchoscopy-Guided Percutaneous Dilatational Tracheostomy Using Conventional versus 3D Printed Simulators (Trac-Sim Study). J. Intensive Care Med..

[12] Jain S., Sayed M.E., Shetty M., Alqahtani S.M., Al Wadei M.H.D., Gupta S.G., Othman A.A.A., Alshehri A.H., Alqarni H., Mobarki A.H. (2022). Physical and Mechanical Properties of 3D-Printed Provisional Crowns and Fixed Dental Prosthesis Resins Compared to Cad/Cam Milled and Conventional Provisional Resins: A Systematic Review and Meta-Analysis. Polymers.

[13] Tejo-Otero A., Buj-Corral I., Fenollosa-Artés F. (2020). 3D Printing in Medicine for Preoperative Surgical Planning: A Review. Ann. Biomed. Eng..

[14] Meyer-Szary J., Luis M.S., Mikulski S., Patel A., Schulz F., Tretiakow D., Fercho J., Jaguszewska K., Frankiewicz M., Pawłowska E. (2022). The Role of 3D Printing in Planning Complex Medical Procedures and Training of Medical Professionals-Cross-Sectional Multispecialty Review. Int. J. Environ. Res. Public Health.

[15] Moldovan F. (2019). Recent Trends in Bioprinting. Procedia Manuf..

[16] Fedorov A., Beichel R., Kalpathy-Cramer J., Finet J., Fillion-Robin J.C., Pujol S., Bauer C., Jennings D., Fennessy F., Sonka M. (2012). 3D Slicer as an Image Computing Platform for the Quantitative Imaging Network. Magn. Reson. Imaging.

[17] Dorweiler B., Baqué P.E., Chaban R., Ghazy A., Salem O. (2021). Quality Control in 3D Printing: Accuracy Analysis of 3D-Printed Models of Patient-Specific Anatomy. Materials.

[18] Wixted C.M., Peterson J.R., Kadakia R.J., Adams S.B. (2021). Three-Dimensional Printing in Orthopaedic Surgery: Current Applications and Future Developments. JAAOS Glob. Res. Rev..

[19] Sun Z., Lee S.-Y. (2017). A Systematic Review of 3-D Printing in Cardiovascular and Cerebrovascular Diseases. Anatol. J. Cardiol..

[20] Sun Z., Liu D. (2018). A Systematic Review of Clinical Value of Three-Dimensional Printing in Renal Disease. Quant. Imaging Med. Surg..

[21] Belykh E., Giovani A., Abramov I., Ngo B., Bardonova L., Zhao X., Loymak T., Mooney M.A., Sheehy J.P., McBryan S. (2021). Novel System of Simulation Models for Aneurysm Clipping Training: Description of Models and Assessment of Face, Content, and Construct Validity. Oper. Neurosurg..

[22] Pearce P., Novak J., Wijesekera A., Loch-Wilkinson T., Redmond M., Winter C., Alexander H., Maclachlan L. (2023). Properties and Implementation of 3-Dimensionally Printed Models in Spine Surgery: A Mixed-Methods Review with Meta-Analysis. World Neurosurg..

[23] Bernhard B., Illi J., Gloeckler M., Pilgrim T., Praz F., Windecker S., Haeberlin A., Gräni C. (2022). Imaging-Based, Patient-Specific Three-Dimensional Printing to Plan, Train, and Guide Cardiovascular Interventions: A Systematic Review and Meta-Analysis. Heart Lung Circ..

[24] Rajzer I., Kurowska A., Frankova J., Sklenářová R., Nikodem A., Dziadek M., Jabłoński A., Janusz J., Szczygieł P., Ziąbka M. (2023). 3D-Printed Polycaprolactone Implants Modified with Bioglass and Zn-Doped Bioglass. Materials.

[25] Qu L., Wang Y., Wang F., Zhang S. (2023). Surgical Outcomes of Percutaneous Endoscopic Lumbar Discectomy in Obese Adolescents with Lumbar Disc Herniation. BMC Musculoskelet. Disord..

[26] Vinas-Rios J.M., Sanchez-Aguilar M., Medina Govea F.A., Von Beeg-Moreno V., Meyer F. (2018). Incidence of Early Postoperative Complications Requiring Surgical Revision for Recurrent Lumbar Disc Herniation after Spinal Surgery: A Retrospective Observational Study of 9,310 Patients from the German Spine Register. Patient Saf. Surg..

[27] Atesok K., Satava R.M., Marsh J.L., Hurwitz S.R. (2017). Measuring Surgical Skills in Simulation-Based Training. J. Am. Acad. Orthop. Surg..

[28] Stroop R., Nakamura M., Schoukens J., Oliva Uribe D. (2019). Tactile Sensor-Based Real-Time Clustering for Tissue Differentiation. Int. J. Comput. Assist. Radiol. Surg..

